# Understanding the Experiences of Rural Community-Dwelling Older Adults in Using a New DVD-Delivered Otago Exercise Program: A Qualitative Study

**DOI:** 10.2196/ijmr.4257

**Published:** 2015-08-13

**Authors:** Arun Agha, Teresa Y L Liu-Ambrose, Catherine L Backman, Jennifer Leese, Linda C Li

**Affiliations:** ^1^ Arthritis Research Canada Richmond, BC Canada; ^2^ University of British Columbia Vancouver, BC Canada

**Keywords:** Otago Exercise Program, DVD, aging, falls, physical therapy, qualitative research

## Abstract

**Background:**

The home-based Otago Exercise Program (OEP) has been shown to reduce the occurrence of falls in community-dwelling seniors. A new OEP DVD was recently developed for people living in rural communities to be used with minimal coaching by a physical therapist.

**Objective:**

This study aimed to understand older adults’ experiences using the DVD-delivered OEP and explore barriers and facilitators to implementing the DVD-delivered OEP from the participants’ perspectives.

**Methods:**

Rural community-dwelling older adults (75 years and older) who participated in a six-month DVD-delivered OEP study were invited to participate in this qualitative study. Two small group interviews were initially conducted to explore the breadth of participants’ experiences with the program. These were followed by semi-structured individual interviews to gain an in-depth understanding of these experiences. An inductive constant comparison analysis of the transcripts was performed. To ensure methodological rigor, field notes, journaling, and an audit trail were maintained, supplemented by peer-review.

**Results:**

Of 32 eligible participants, five participated in group interviews and 16 in individual interviews. Three themes emerged. Theme 1, The OEP DVD—useful training tool but in need of more pep, represented participants’ experiences that the DVD provided important guidance at program onset, but was too slow and low-energy for longer-term use. Theme 2, Gaining control over one’s exercise regimen, but sometimes life gets in the way of staying active, described participants’ appreciation of the program’s flexibility, but personal health concerns and everyday lives posed challenges to adhering to the program. Theme 3, Social creatures—wanting greater human connection during exercise, described how some participants desired further social interactions for enhancing motivation and receiving guidance.

**Conclusions:**

Individuals should be encouraged to refer to the OEP user manual or DVD as needed and engage friends and family in exercises. The importance of exercise even when living with health problems should be raised at program onset, and participants should be supported in working through challenging issues. Health professionals should work with individuals to integrate the program with their everyday activities.

## Introduction

### Background

In 2014, 15.6% (more than six million) of the Canadian population was aged 65 years or older [1]. By 2030, it is estimated that seniors will constitute 23% (more than 9.5 million) of Canadians [1]. Aging is related to decreased muscle mass and strength as well as declined cognitive speed and memory performance [2-7]. Balance control also decreases during aging, likely due to reduction in muscle strength and joint range of motion, increased reaction time, and changes in sensory systems [8]. Myers et al [9] and Carter et al [10] reported that a decrease in muscle strength, diminished cognitive function, and impaired balance in older adults increases the risk of falls. Approximately 30% of all community-dwelling seniors experience one or more falls every year [11-13]. Falls are estimated to cost the Canadian health care system Can$2 billion annually (in 2004 dollars) [14]. Almost half of all individuals who have had a fall experience a minor injury, and 5% to 25% experience a more serious injury like a fracture or sprain [15]. Most falls in community-dwelling older adults are due to slips and trips [16-18]. It is proposed that such falls occur due to a person’s inability to regain balance [16].

Physical activity and exercise promote healthy aging [8,19-28]. Resistance exercise has been shown to mitigate muscle fiber loss and improve balance and cognitive function [8,19-23,27-29]. Several studies have reported that resistance exercise combined with balance exercise could prevent falls in older adults [28,30-33]. It is thought that exercise accomplishes this by ameliorating specific age-associated physiological changes that act as risk factors for falls, including reduced muscle strength, impaired balance, and diminished cognitive function [9,10]. Promoting physical activity is specifically important among older adults living in rural communities because of the prevalence of physical inactivity and the greater likelihood of falls [34-37].

### The Otago Exercise Program

**Figure 1:**
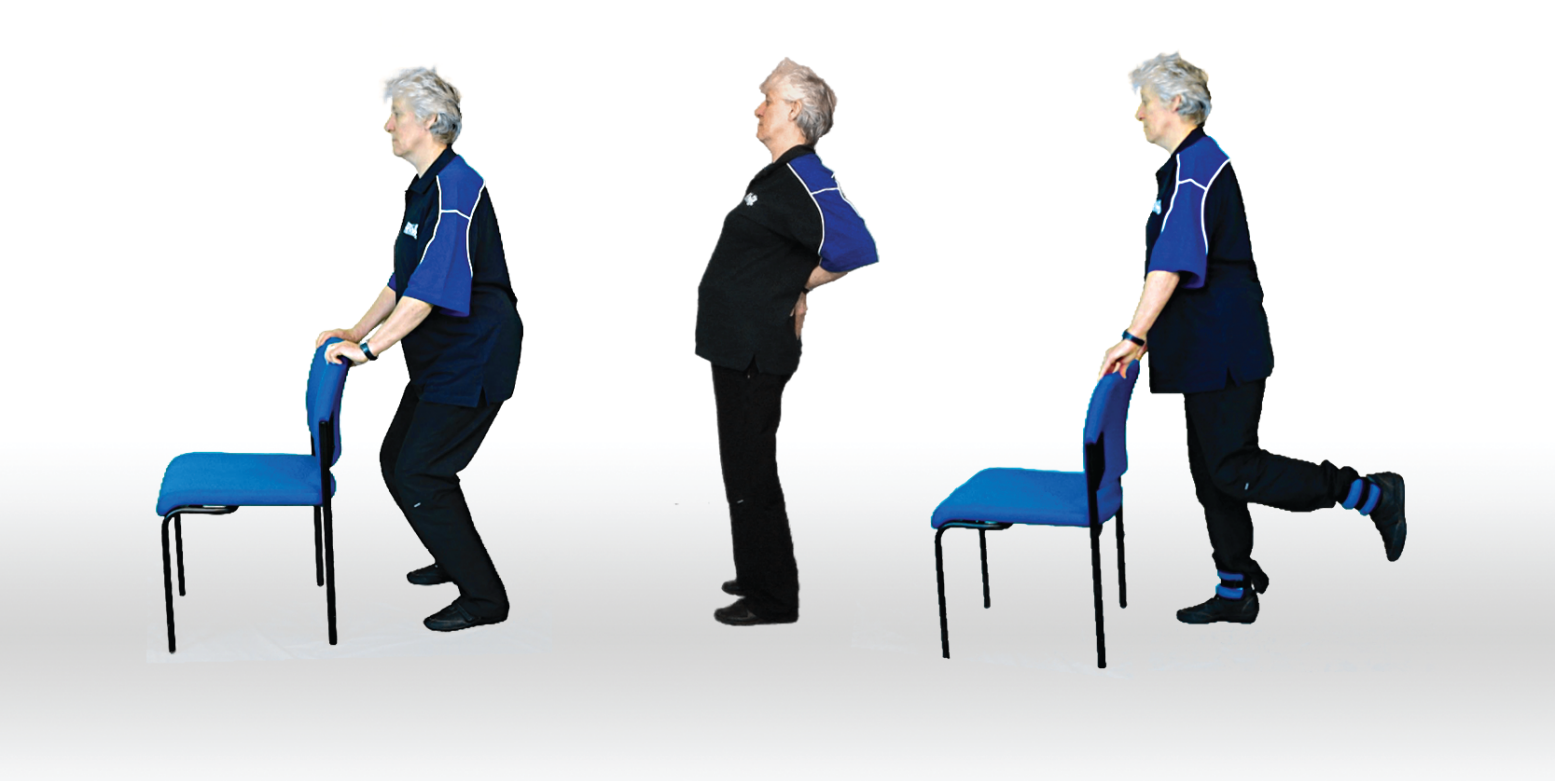
Screenshot of strength and balance exercises from the Otago Exercise Program DVD.

The Otago Exercise Program (OEP) was developed by Campbell and Robertson [28] in 1997 to prevent falls in older adults. This program consists of individually tailored exercises intended to improve strength and balance in seniors [28,30] (Figure 1). The exercises aim to strengthen lower limb muscles (eg, knee extensors and flexors, hip abductors, and ankle plantarflexors and dorsiflexors) and improve balance (eg, balance retraining exercises include knee bends, backwards walking, walking and turning around, sideways walking, tandem stance and walk, one leg stand, heel walking, toe walking, heel-toe walking backwards, and sit-to-stand activities). The OEP is designed to be performed at least three times a week for 30 minutes [28]. Participants are also encouraged to take short walks at least twice a week. The program is normally delivered by a physical therapist who makes four or five home visits with the individual over six months. During the initial visit, the physical therapist conducts a physical assessment on the individual and prescribes exercises from the instructional OEP booklet. Participants then receive a copy of the exercise booklet, ankle cuff weights, and a calendar to record exercise and fall frequency. The physical therapist makes progressive adjustments to the exercises in the follow-up visits.

Robertson et al [38] conducted a meta-analysis of the OEP using individual level data from the initial four OEP controlled trials [28,30-32,39] with a total of 1016 community-dwelling seniors. The results showed a 35% reduction in falls and fall-related injuries when comparing the exercise groups against the control groups (falls: incidence rate ratio [IRR] 0.65, 95% CI 0.57-0.75; fall-related injuries: IRR 0.65, 95% CI 0.53-0.81). The program was more effective in reducing injurious falls for individuals aged 80 years and older compared to younger individuals (IRR 0.54, 95% CI 0.34-0.87). The fall-reducing benefits were equal for older adults with and without previous falls and for women and men. Approximately 70% of participants in the OEP group were engaging in some exercise at the end of the studies. In a one-year randomized controlled trial, Liu-Ambrose et al [40] evaluated the effectiveness of the OEP in 74 community-dwelling seniors in British Columbia, Canada. After one year, the adjusted IRR of falls in the OEP group compared to the guideline care control group was 0.47 (95% CI 0.24-0.96). Gillespie et al [41] reported in a Cochrane systematic review that individual home exercise programs for community-dwelling older adults that incorporated two or more components of strength, balance, flexibility, or endurance exercises reduced the rate of falls (IRR 0.66, 95% CI 0.53-0.82) and the number of individuals falling (IRR 0.77, 95% CI 0.61-0.97). The review concluded that there was strong evidence supporting the use of the OEP for fall reduction among older adults. Suggested plausible mechanisms may include improvements in executive functioning, balance, and/or lower body strength.

### Program Delivery in Rural Communities

Evidence supports the use of the OEP for reducing fall occurrences in older adults. It is not always feasible, however, to deliver the program in its current state in some rural communities because it requires multiple home visits by a physical therapist, and there is a shortage of health professionals, including physical therapists, in rural Canada [42]. Although 19% of the Canadian population lives in rural areas (Statistics Canada, 2011 Census of Population), only 8% of physical therapists work in rural or remote communities [43], so they usually have a heavy caseload. Due to these challenges, alternatives to the four to five physical therapist home visits are required in order to sustain the delivery of the OEP program in a rural setting. DVDs are a viable option for delivering health and lifestyle interventions. The sale of exercise DVDs experienced an annual growth of 11.2% from 2008 to 2012, with over 20% of them purchased by older adults [44]. Past studies suggest that adherence to video-based exercise programs can increase muscle strength [45,46], physical function [46,47], health-related quality of life [48], and psychological well-being in older adults [49]. Average adherence rates in these studies was 58% to 100% [45-49].

Liu-Ambrose et al recently developed and tested a new method of delivering the OEP to older adults in rural communities [50]. This pre-post study evaluated a new method that involved one in-person visit with a physical therapist, who performed an assessment and prescribed specific exercises from a new OEP DVD. The OEP DVD consisted of narrated video footage of a model performing the various exercises. The footage aimed to instruct, inform, and motivate the user. Prior to demonstrating each exercise, introductory narrated footage described the muscle(s) the exercise targets and its potential affects on physical function. This was followed by detailed instructions on how to perform the exercise. The physical therapist followed up with individuals monthly by telephone for six months to adjust their exercise program. Individuals also received an exercise manual, ankle cuff weights, and a program calendar.

We embedded a qualitative enquiry within the evaluation of this new method of delivering the OEP. The purpose was to understand older adults’ experiences in using the DVD-delivered OEP and to explore the barriers and facilitators to implementing this new method of OEP delivery.

## Methods

###  Study Design

This qualitative study is informed by the principles of grounded theory as outlined by Glaser and Strauss [51]. Grounded theory is a methodology that “uses iterative data collection (such as interviews, observations) and analysis to build theories about social phenomena” [52]. While generating a theory was not the goal of this study, an understanding of participants’ experiences was the intended outcome. The knowledge generated using this approach is developed inductively from the empirical data obtained in naturalistic settings.

### Participants

A total of 32 people living in Sechelt and Gibsons, two rural communities in British Columbia, volunteered to participate in the six-month OEP evaluation (the main study). Individuals were eligible if they (1) were 75 years or older, (2) were community-dwelling (ie, not residing in a nursing home, extended care unit, or assisted care facility), (3) scored 24 or higher in the Mini-Mental State Examination (MMSE), (4) were capable of walking 100 meters independently (with or without assistive tools), (5) owned a working DVD player and television, and (6) were able to provide informed consent. The study excluded people who had (1) a previous diagnosis of a neurodegenerative disease, (2) a previous diagnosis of dementia, (3) a clinical stroke, or (4) a history indicating carotid sinus sensitivity (ie, syncopal falls). All participants from the main study were invited to partake in the qualitative study.

### Qualitative Study

This qualitative study was composed of initial group interviews followed by in-depth individual interviews. The purpose of the groups was to explore the breadth of topics brought forth by participants and identify topics for further in-depth exploration by individual interviews. For the individual interviews, participants had the option of face-to-face or telephone interviews. During data collection, we recorded field notes and reflections. This study received ethics approval from the University of British Columbia (H11-01604) and the Vancouver Coastal Health Authority.

### Data Analysis

Following each interview, audio recordings were transcribed verbatim and verified. NVivo 10 software (QSR International) was used for data organization and analysis. An inductive constant comparison analysis was performed [53]. Each transcript was read and codes were assigned to sentences, paragraphs, and sections [53]. Codes were generated from the data and were not pre-determined. After initial coding, the data were constantly revisited until saturation was achieved [53]. Similarly coded sections were compared against each other to ensure consistency in the definition of the codes [54]. Codes with similar elements were combined to form categories [54]. These categories were then clustered around the research questions they contributed to answering, and broad themes and patterns were identified [53]. This analytical process was recorded and an audit trail was maintained. The first author (AA) led all analyses, and team members queried main categories and justifications. One investigator (LL) and one volunteer team member (MK, see Acknowledgements) reviewed four individual interview transcripts to enhance credibility by detecting issues in the analysis such as overemphasized or underemphasized points, vague descriptions, and assumptions made by the researchers.

## Results

### Participants

Between April 2013 and January 2014, 21 OEP main study participants took part in the qualitative study (five participants in group interviews and 16 participants in individual interviews). The majority were female. Participants varied in age from 74 to 97 years, and half had some post-secondary education. Table 1 presents demographic characteristics of participants who completed group and individual interviews.

Eleven OEP main study participants did not participate in the interviews; of those, six were men. The age of the 11 individuals varied from 78 to 89 years, and six had a high school certificate or diploma as their highest level of education.

The majority of participants found the OEP to be useful and reported that the program positively impacted their physical and mental health. Participants felt that the OEP improved their balance and lower body strength, which some believed alleviated their fear of falling. Living with chronic health conditions acted as a significant barrier to engaging in certain activities, including domestic chores and exercise. Participants spoke about living very busy lives and managing their limited time to fulfill all their commitments. Nevertheless, most participants planned to continue performing the OEP in some capacity to further reap the benefits of the program.

**Table 1:** Characteristics of participants who completed group and individual interviews.

Interview types	Pseudonyms	Sex	Age	Body mass index	Number of concurrent medications	Highest level of education	Interview method
**Pilot group interviews**							
	Anne	Female	74	22.0	1	High school certificate or diploma	Face-to-face (group interview #1)
	Betty	Female	75	24.4	0	Some university without certificate or diploma	Face-to-face (group interview #1)
	Claire	Female	82	21.2	2	University degree	Face-to-face (group interview #1)
	Dana	Female	85	28.1	0	Grades 9-13, without certificate or diploma	Face-to-face (group interview #2)
	Edna	Female	77	25.2	2	University degree	Face-to-face (group interview #2)
**In-depth individual interviews**							
	Frank	Male	78	32.3	0	High school certificate or diploma	Face-to-Face
	Gabby	Female	81	29.1	3	University certificate or diploma	Face-to-Face
	Holly	Female	81	28.8	2	Some university without certificate or diploma	Face-to-Face
	Ida	Female	82	30.1	11	High school certificate or diploma	Telephone
	Jenny	Female	88	28.0	0	Trades or professional certificate or diploma	Telephone
	Katy	Female	81	21.3	3	High school certificate or diploma	Telephone
	Lacy	Female	75	29.5	1	High school certificate or diploma	Telephone
	Mary	Female	97	30.8	4	Trades or professional certificate or diploma	Telephone
	Ned	Male	90	30.6	0	Grades 9-13, without certificate or diploma	Telephone
	Oliver	Male	77	32.2	2	University degree	Telephone
	Patty	Female	82	31.3	0	Grades 9-13, without certificate or diploma	Telephone
	Quinn	Male	84	25.0	2	High school certificate or diploma	Telephone
	Roberta	Female	77	24.4	3	University degree	Telephone
	Sandra	Female	82	26.7	3	University certificate or diploma	Telephone
	Thomas	Male	84	31.4	3	Trades or professional certificate or diploma	Telephone
	Walter	Male	79	27.9	3	High school certificate or diploma	Telephone

### Qualitative Themes

#### Theme 1: The OEP DVD—Useful Training Tool but in Need of More Pep

In general, participants stated that the DVD presented a good overview of the OEP.

I learned the correct posture and positioning…of the exercise[s] [from the OEP DVD]…

Edna, group interview #2

One key critique of the OEP DVD was that it was slow and long and in need of more pep. This lack of pep seemed to be due in part to the muted music and lack of energy in the exercise demonstration. Some disliked the slow pace of the exercise instructions and found the inability to skip the introductory video footage of each exercise frustrating. These issues became especially bothersome as participants became more experienced with the program.

I tried very hard to do the exercises the way they were demonstrated in the video. But…as I got to know the sequence of the exercises, it…extended the exercise time too much…so I dispensed with the video…

Oliver

Several participants said they wanted a version of the DVD that had a faster pace and more upbeat music. On the other hand, some simply replaced the DVD with the written manual after several sessions because it was more convenient to use.

you could have shown [the exercises] slowly at…first, but then if you want us to use [an] exercise DVD [during the program] you should have had [another version] that went a little bit faster

Lacy

it wasn't practical to do the DVDs…[The manual] was quite good because it was quite clear and well-illustrated and you could see what had to be done and so on. So it was quite easy to follow it…

Thomas

#### Theme 2: Gaining Control Over One’s Exercise Regimen, but Sometimes Life Gets in the Way of Staying Active

Participants appreciated the flexibility of the program. The home-based design of the program allowed participants to choose convenient and preferred exercise times and locations.

you weren't tied down…[It] didn't have to be…Monday, Wednesday, Friday. [It] could be Monday, Tuesday, and Friday you know, whatever. That was a good aspect of [the program]

Thomas

Some integrated the exercise program into their daily routines, such as exercising in between household chores, while reading or watching TV, or while taking walks. This enhanced their feeling of flexibility and control over the program.

after dinner clean up…I might be sitting for two, three hours in the evening with a book or TV but during that time I’ve been using the leg weights

Katy

Participants contrasted the flexibility of the OEP with the travel barriers and time demands they encountered with center-based exercise programs. These barriers were often the result of preexisting medical conditions, inconvenient travel, or scheduling demands.

A lot [of] older people my age can’t drive. Their vision, I can’t drive at night…and a lot of exercise programs are in the evenings for people after work but that doesn’t apply to seniors. But any one that was in the evenings in the wintertime forget it, I couldn’t drive because [of the] headlights coming at me, I have glaucoma and I just can’t drive at night

Holly

Participants also did not like being dependent on others when attending center-based exercise programs (eg, being dependent on transportation) and sometimes felt uncomfortable in such settings (eg, being in groups with mostly younger people).

One participant discussed how she accommodated the OEP to her health conditions (Jenny and her incontinence). For the majority of participants, however, preexisting chronic conditions (eg, arthritis) as well as depression and postexercise pain and fatigue were cited as barriers to continuing the OEP.

I haven’t been walking [in the OEP]…because of these hips and I’ve also developed a problem with a couple of spots on the bottom of my feet…

Katy

the [OEP exercise of] going up and down stairs…I find that difficult and I don’t know that I could really improve…[because of] the lower back problems I have

Ned

the word depression comes into it but…[the program] just seems like not a priority

Frank, discussing the OEP

Some participants also found it challenging to maintain the OEP routine due to other events and activities in their lives. For example, Katy’s renovation of her home and her subsequent illness made it difficult to exercise. Other participants had different priorities (eg, taking care of ill loved ones) and felt they had insufficient time to perform the program.

Setting aside the time [made it difficult to use the program]…‘Cause I may be retired, but I’ve still got a lot of stuff to do

Walter

Several OEP exercises required ample safe walking space and balancing support. For some participants, it was difficult to find this in their homes or neighborhoods. For instance, Mary was not able to use the OEP DVD because the DVD player was in a room with insufficient space to perform certain exercises. Gabby described not being able to walk during the wintertime due to harsh weather and a fear of falling.

#### Theme 3: Social Creatures—Wanting Greater Human Connection During Exercise

In the context of this study and based on the transcripts in their entirety, we define social creatures as individuals who appreciate human connections to guide and motivate them to be active. Such connections included interactions between peers in an exercise setting as well as health professional encounters. Some participants felt they would be more motivated or feel more energetic if they were in a group OEP involving peers and live instructors, where they could feed off others’ energy and ensure proper exercise techniques.

I like group work, sharing it with somebody and you can compare what you’re doing to what they are doing…It’s the same way if you’re taking art lessons…you can see how you compare to the next fellow

Jenny

Similar to the DVD, participants described the program as a whole as becoming boring and repetitive as they became familiar with the exercises. For example, Jenny thought the program became tiresome because of the repetitiveness of the exercises and lack of social contact.

sitting…by yourself in your house and doing it just gets [too] much after six months…I found it a real drag at the end…repeating the same thing night after night…

Jenny

Some participants believed that a group exercise setting might have lessened these feelings of boredom by immersing participants in a social setting. For instance, Betty felt that placing the OEP in a social setting would have made the program more entertaining because of the peer interactions. Additionally, Holly explained that her past experience in the Jazzercise exercise program, where she was interacting with others in a social setting with music, created a fun environment, and she felt that the OEP in a similar setting would have been more fun.

you’re doing [the OEP] on your own… [if you would be] with a group at the activity center…[it would be] much more fun because you’re with other people and you know the instructors, it’s more like Jazzercise used to be, I used to do that years ago and it was fun and the music is good and it’s got a good beat to it and it gets you going. And this one with you being on your own and no music to speak of, I mean it just had sort of a rhythm in there, just a baseline…

Holly

Some participants also enjoyed the camaraderie when exercising in a group setting. Several participants felt that the OEP would benefit from adding a group exercise option to the home-based program. For example, Patty says,

I think that if there was a facility once a week, say, at the [community] center…if it was offered once a week, it would be a growing thing for seniors. Yes, [like] a group program… I think something like that, I would certainly go to, because it’s so beneficial

Patty

Not all participants agreed, however, with the idea of adding a group component to the OEP. Several cited transportation as a major barrier while others preferred to exercise alone and with minimal instructions. For these participants, it appeared that the home-based program was preferred over an exercise group.

I don't think [performing the OEP strength and balance exercises and walks with other people would] make much difference to me because I’ve done most of my exercises alone for many, many years. So I don't think it would change my energy or enthusiasm for exercising. You know, I know that exercise is good for me, so I try to do it.

Oliver

Participants also felt that the guidance and motivation received from the physical therapist were beneficial and some would prefer more home visits to assess their performance rather than the telephone follow-up.

I think that six months is a long time to have people doing things on their own…the phone calls are fine, but I think perhaps a visit here and there might have been a helpful thing…I would suggest that [to] keep people motivated…

Sandra

Despite wanting further physical therapist interaction, participants seemed to be aware of the resource challenges present in many rural communities, including their own, and felt that such additional interactions were not always feasible. Participants generally felt that the walking component of the OEP was fairly easy to complete, with several participants walking more than was necessary per program guidelines and some already walking prior to starting the program. Some made the walking more social by walking with friends or family members.

## Discussion

### Principal Findings

The purpose of this study was to understand older adults’ experiences using the DVD-delivered OEP and explore the barriers and facilitators to implementing this new method of OEP delivery. Three themes emerged from the analysis of the interviews. The first theme represents how the OEP DVD provided participants with important guidance at program onset but was too slow, long, and low-energy for continued use. The second theme outlines participants’ appreciation of the flexibility of the program, but their own health, their loved ones’ health, and other activities in their lives sometimes make it challenging to exercise. The third theme describes participants’ beliefs about the benefits of social interaction in exercise programs like the OEP to maintain motivation and long term adoption of exercise habits.

Our findings suggest that existing health conditions associated with pain and fatigue may be a barrier for older adults to engage in an exercise program. This is in line with the literature [55-60]. In the Belza et al [55] qualitative study of 71 underserved, ethnically diverse older adults, barriers to physical activity included physical conditions, such as hearing or visual impairments and feeling tired or dizzy, and psychological conditions. Cohen-Mansfield et al [56] surveyed 324 community-dwelling older adults and found that the most common self-reported barriers to exercise were health problems such as depression and pain. Those authors suggested the need to address pain and depression and stress the importance of exercise even when living with health problems to increase older adults’ exercise participation. Programs like the OEP may be enhanced by integrating recommendations for accommodating adverse symptoms into the exercise instructions.

Participants largely felt that an OEP group, if they had the chance to access one, might have offered additional motivation to exercise. This supports other studies suggesting that social interactions and support are important facilitators of exercise in older adults [56,60-62]. In a qualitative study involving older adults, Stathi et al [62] identified social interaction as a reason for people attending group exercise sessions because they enjoyed connecting with others and expanding their social network. Fox et al [61] also demonstrated that older adults’ relationships with exercise instructors and peers facilitated their participation in an exercise program. Specifically, older adults felt that the group setting provided a friendly and supportive environment that offered socializing opportunities.

Social isolation is a major concern for older adults in rural communities. In a study by Paluck et al [63], older women living in a rural community spoke about the adverse impact that social isolation and loneliness had on their efforts to remain healthy, including engaging in exercise. Mutual support of peers in group settings could be a means to maintaining a physically active lifestyle in a rural community [63]. The themes generated in the current study are in line with a recent thematic synthesis of 132 qualitative studies involving 5987 participants on older adults’ perspective on participation in physical activity [64]. Particularly, social influences (eg, valuing interaction with peers, encouragement from others, dependence on professional instruction); physical limitations (eg, pain or discomfort, comorbidities); competing priorities (eg, work and family responsibilities); access difficulties (eg, environmental barriers including weather and transportation); personal benefits of physical activity (eg, strength, balance and flexibility, self-confidence, independence, improved health and mental well-being); and motivation and beliefs (eg, apathy, irrelevance and inefficacy, maintaining habits) were themes identified that resonated with our findings. It appears that an exercise program with DVD demonstration and a clear manual in combination with one or two physical therapist visits and some optional community support may be an option to be explored in any community, rural or urban, in order to deliver proven fall-prevention exercise interventions.

###  Limitations and Strengths

This study has several limitations. All participants were individuals who completed the six-month OEP evaluation study. Individuals who did not meet a certain level of cognitive functioning (ie, score 24 or higher in the MMSE) or who had a clinical stroke or diagnosis of a neurodegenerative disease were excluded from the main study. This limits the transferability of the findings to older adults with characteristics similar to those in this qualitative inquiry [65]. Because most of the individual interviews took place over the telephone, the depth of the observational data collected was limited. For example, we could not observe nonverbal communications or body language. Moreover, data collection occurred throughout a 10-month period (April 2013 to January 2014). As such, several factors, including seasonal changes and holidays, might have influenced participants’ experiences with the exercise program. For example, the cold and rainy winter in British Columbia might affect people’s participation in outdoor activities such as walking. Such aspects might not have been fully explored during the interviews. Despite the limitations, the inductive nature of this study facilitated the emergence of findings that were grounded in the lived experience of participants in rural communities. Moreover, we included strategies such as peer review, field notes, journaling, and an audit trail, which have been shown to improve rigor and trustworthiness.

### Conclusions

This study’s results construct an understanding of the experiences of older adults living in a rural community in using the new DVD-delivered OEP. Insight was gained on the facilitators and barriers to partaking in this program from the participants’ perspectives. First, the versatility and convenience of the written manual might make it easier for participants to complete the OEP. Next, the home-based design of the OEP provided the flexibility and sense of control over when and where to exercise, allowing participants to better integrate exercise into daily routines. In addition, social support from family and friends was identified by participants to facilitate adherence to the OEP. In terms of barriers, personal health concerns were a key hurdle to performing exercise. This finding supports the importance of highlighting the benefits of resistance exercise and physical activity for people with a variety of health conditions as a way to encourage older people to adopt and maintain an active lifestyle. Moreover, individuals might prioritize certain other obligations and activities over exercise; thus, in addition to personal health concerns, everyday responsibilities or activities should be discussed prior to the start of the program and methods of integrating the program with these activities should be promoted (eg, promoting the versatility of the written manual). Finally, the boredom participants experienced in the OEP once they were familiar with the routine could be ameliorated by including a greater level of social interaction during exercise. It may be useful to recommend to participants to perform parts of the OEP with family members or peers. By identifying the facilitators and barriers, this study provides the necessary background for the implementation of the DVD-delivered OEP in other rural communities.

## Abbreviations

IRR: incidence rate ratio
MMSE: Mini-Mental State Examination
OEP: Otago Exercise Program
